# Regulatory Genes as Beacons for Discovery and Prioritization of Biosynthetic Gene Clusters in *Streptomyces*

**DOI:** 10.1021/acs.biochem.4c00711

**Published:** 2025-03-26

**Authors:** Hannah E. Augustijn, Daan van Nassauw, Simona Cernat, Zachary L. Reitz, Gilles P. van Wezel, Marnix H. Medema

**Affiliations:** 1 Bioinformatics Group, 4508 Wageningen University, Wageningen 6708 PB, The Netherlands; 2 Molecular Biotechnology, Institute of Biology, 4496 Leiden University, Leiden 2333 BE, The Netherlands

## Abstract

Actinobacteria are renowned for their ability to produce a wide range of bioactive molecules, including many anticancer compounds and antibiotics that are critical in the battle against antimicrobial resistance. Despite identification of a vast array of biosynthetic gene clusters (BGCs) through genome mining, much of this biosynthetic potential remains unexplored, partially due to the fact that many remain silent or cryptic under typical laboratory conditions. Regulatory networks can provide clues to the location of yet undiscovered gene cluster families or be leveraged to predict their expression. Here, we investigate the associations between regulatory genes and BGCs to uncover their predictive capabilities in discovering and prioritizing gene clusters for downstream wet-lab validation. By analyzing the protein domain architectures of 128,993 potential regulators derived from 440 complete *Streptomyces* genomes, we uncovered various associations between biosynthetic classes, biological activities of their products, and regulator families. Specifically, subsets of the *Streptomyces* Antibiotic Regulatory Protein (SARP) and LuxR families were strongly associated with biosynthetic pathways encoding the production of bioactive compounds. After closer genomic inspection of the small SARPs, we discovered 82 putative SARP-associated BGCs that escaped detection by state-of-the-art software. This shows that continued exploration of regulatory systems will not only deepen our understanding of Actinobacteria’s biosynthetic capabilities but also facilitates discovery and prioritization of high-potential BGCs in future genome-mining applications.

## Graphical abstract

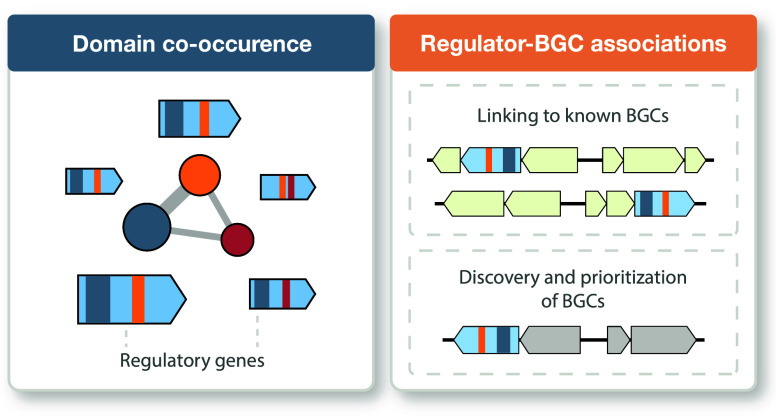

## Introduction

The introduction of antibiotics as clinical agents has drastically changed prevention and treatment of infectious diseases, saving millions of lives worldwide. However, the mass production and extensive use of these valuable natural products have created unprecedented selection pressures, causing the spread of resistance among bacteria and the emergence of multidrug-resistant strains.^1,2^ Simultaneously, the success rate of traditional antibiotic development through high-throughput screening has dramatically declined, pointing to the need for innovative approaches to discover novel metabolites with clinical potential.^3,4^ As producers of two-thirds of all clinically used antibiotics and numerous other medically relevant bioactive molecules, members of the phylum Actinobacteria are a prime source of specialized metabolites.^5,6^ Their potential lies hidden within their genomes, which contain sets of colocalized genes typically encoding enzymes that act within the same biosynthetic pathway, known as biosynthetic gene clusters (BGCs).^7^ Recent advances in genome sequencing and computational methods such as artificial intelligence have enabled scientists to identify numerous biosynthetic genes and gene clusters,^8–10^ although only a fraction have been experimentally characterized, as many remain silent or are sparingly expressed under standard laboratory conditions.^11,12^ This is mainly due to our limited knowledge and inability to replicate the environmental stimuli that trigger the host’s native regulatory system, which in turn controls the expression of BGCs and production of metabolites.^13^

To characterize this unexplored potential, researchers have developed several methods that introduce such stimuli in the laboratory, including varying strain-cultivation conditions, cocultivation, or high-throughput elicitor screening strategies.^14–16^ Despite these efforts, the success rate of random screening for bioactive compounds remains low, primarily because of the frequent rediscovery of known compounds.^17^ Additionally, the sheer number of predicted BGCs makes it difficult to identify and prioritize those with the potential to become novel drug targets. This challenge, in turn, complicates the use of more targeted approaches, such as heterologously expressing BGCs in alternative hosts.^18^ Since regulation is crucial for the transcription of BGCs, addressing these challenges can benefit from a deeper understanding of this, in order to aid effective prioritization of promising drug targets as well as elicitation of expression of BGCs in their native hosts.

Various strategies have been developed to harness the regulatory machinery to address these challenges.^19^ These approaches typically focus on regulators, typically transcription factors (TFs), which together form complex regulatory networks. These networks are controlled by both global (pleiotropic) regulators that control numerous targets, and cluster-situated regulators (CSRs), which are believed to influence the expression of genes within specific pathways.^20,21^ CSRs, in particular, have gained attention for their potential to directly regulate BGC expression, which is of interest not only for insights into how to trigger expression, but also for prioritizing BGCs for experimental validation. For instance, regulators from the *Streptomyces* antibiotic regulatory protein (SARP) family are well-known CSRs of antibiotic BGCs and are often considered markers for promising drug targets.^22–24^ However, the presence of CSRs in BGCs and their association with biological activities or biosynthetic classes has not been comprehensively and quantitatively studied or characterized. Even within the SARP family, there are subtypes, and some are believed to be more frequently associated with antibiotic BGCs, although this has yet to be systematically explored.^25^

In this work, we provide a comprehensive analysis of the domain architectures of regulators and their associations with BGCs to identify markers for potential novel or relevant chemistry and bioactivity. By providing a systematic overview of the most common regulatory types in *Streptomyces* species, the model organism of Actinobacteria, and associating these with predicted BGCs, we identified 11 regulatory subgroups that have a preferential association with BGCs compared to other genomic regions. Further exploration using SARP- and LuxR-type regulatory subclasses as genomic markers for discovery allowed us to pinpoint 82 and 86 regions with predicted new biosynthetic potential. Taken together, these findings not only enhance our understanding of the regulatory system but also demonstrate the method’s effectiveness in prioritizing promising gene regions for experimental validation, potentially contributing to acceleration of the discovery process of novel bioactive molecules and drug candidates.

## Results

### Mapping the Regulatory Protein Family Landscape in *Streptomyces*

To obtain insights into the distribution of genes encoding CSRs relative to BGCs, we first generated a comprehensive overview of regulatory proteins in *Streptomyces* species. For this, all protein-coding regions potentially encoding transcriptional regulators were extracted from 440 complete *Streptomyces* genomes. This collection included all completely assembled genomes available as of August 2023, ensuring that no genes or BGCs were missed due to their location at contig edges (Table S1). This identified 128,993 putative regulators corresponding to a collection of 1375 regulatory-associated PFAM domains. Next, we identified the domain composition of each putative regulator to classify them into subgroups of related regulatory proteins. Given that different proteins often share common domains, we analyzed the co-occurrence of specific domains within individual proteins. The resulting data were visualized as a domain co-occurrence network (Figure 1A). In this network, each node represents a unique protein domain, and edges indicate co-occurrence of domains within the same protein. The most frequently occurring domains were TetR_N (PF00440, *n* = 46,647), HATPase_c (PF02518, *n* = 36,759), and Response_reg (PF00072, *n* = 31,031) (Table S2). From the network, we identified several regulatory protein families. The most common families are labeled in Figure 1A, with histidine kinases (*n* = 49,215) and the TetR family (*n* = 48,005) being the most prevalent, followed by (anti)­sigma factors (*n* = 18,822), the LuxR family (*n* = 16,183), and the LysR family (*n* = 13,444).

**Figure 1:**
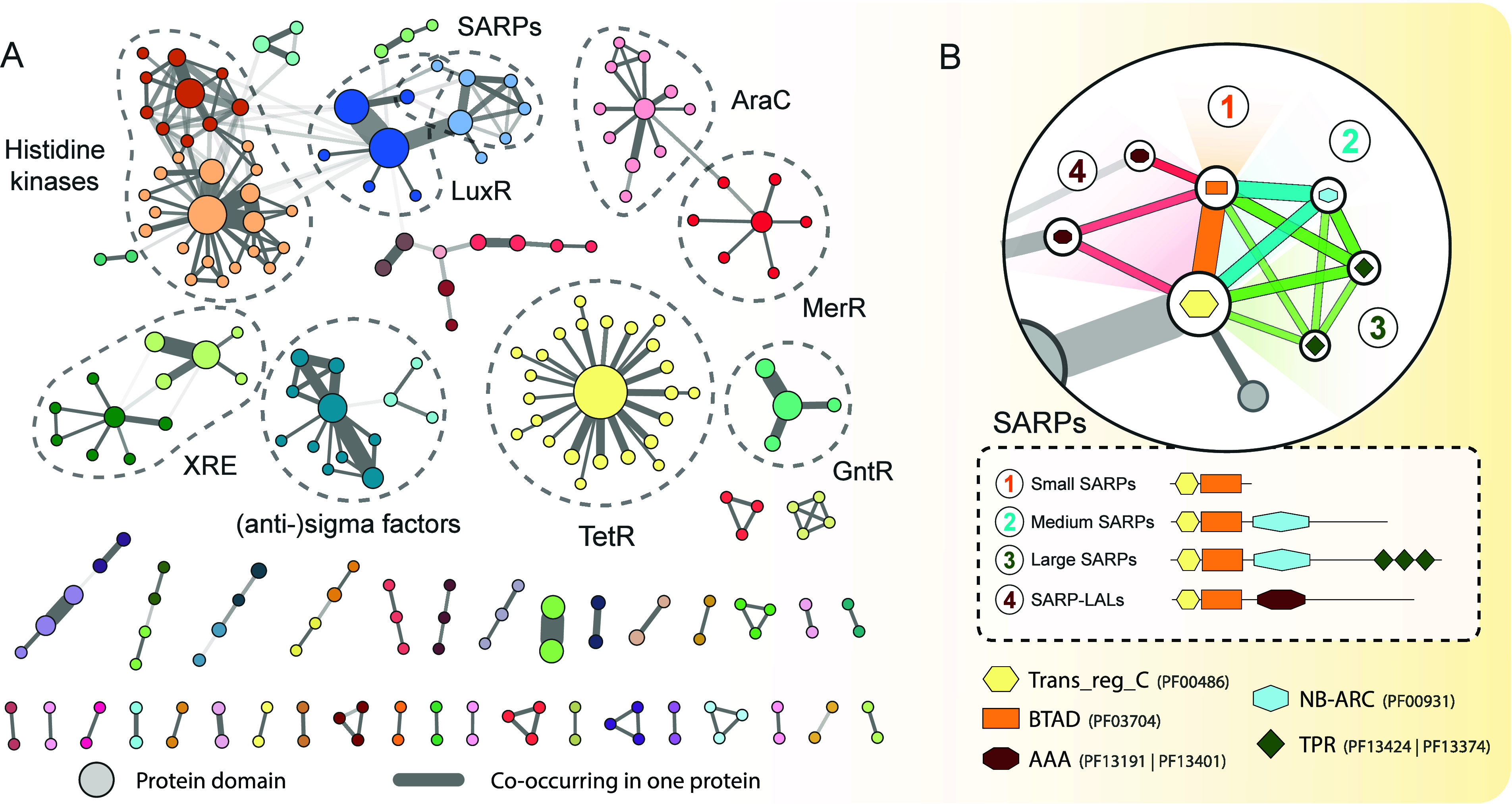
Regulatory domain co-occurrence network and subclustering of SARP regulators. (A) Regulatory domain co-occurrence network in *Streptomyces* genomes. Each node in the network represents a unique PFAM domain, with node size proportional to the frequency of its occurrence in the data set. Nodes are connected by edges if their respective PFAM domains co-occur within a single protein. The width of the edges indicates the frequency of these co-occurrences, while edge transparency reflects the proportion of times the smaller node co-occurs with the larger one, highlighting instances where the smaller domain consistently co-occurs with the larger domain. Subclustering within the network was performed using the Markov Clustering Algorithm (MCL) using the frequency of co-occurrence as weight. The complete Cytoscape network file can be downloaded from the supplementary data files. (B) Subclustering of SARP family regulators. A detailed view of the subclustering within the SARP family, illustrating the different subclasses identified based on domain composition.

To identify which regulatory genes might be associated with BGCs, we further subdivided the families into subclasses through manual curation (Figure 1B). Consistent with existing literature,^22,25^ SARP family regulators could be divided into four distinct subclasses: (1) small SARPs, which consisted solely of the transcriptional regulatory protein C-terminal (Trans_reg_C, PF00486) and the bacterial transcriptional activator domain (BTAD, PF03704); (2) medium-sized SARPs, which also contain the NB-ARC (PF00931) domain; (3) large SARPs, primarily characterized by one of two types of tetratricopeptide repeats (TPR_10, PF13374 and TPR_12, PF13424); and finally, (4) a combinational subclass of SARPs and a LuxR family type (LAL) that contains AAA ATPase domains (AAA_16, PF13191, or AAA_22, PF13401) instead of tetratricopeptide repeats. This process was repeated for all possible combinations within the network. See Table S3 for a complete overview of these combinations.

### Identifying Functional Relationships of Regulatory Gene Families with BGCs

Next, we explored associations between regulatory genes and BGCs to identify which (sets of) regulatory protein families show associations with natural product biosynthetic classes or biological activities. We evaluated the ratio of regulatory genes located inside versus outside of antiSMASH-predicted BGC regions for all domain combinations (Figure S1A). On average, 14.2% of regulatory genes were found within predicted BGC ranges, which, given that 14.6% of coding regions fell within these ranges, is similar to the frequencies of genes in general (Figure S2B). Therefore, we focused on regulatory subclasses in the upper quartile of the association, identifying 12 subclasses that appear to be more closely linked to BGCs. These included genes for TetR family regulators (single domain TetR_C_33, PF13305), genes for three subclasses of LuxR regulators (AAA_16, small-sized, and large-sized PAS-LuxRs), genes for three subclasses of SARP-family regulators (small-sized, medium-sized, and SARP-LAL), as well as genes for LitR-, XRE-, ScbR-, NrdR-, and LexA-family regulators. For each of these subclasses, we identified the BGC class of the closest core gene to determine relationships between regulatory genes and certain BGC types (Figure 2A).

**Figure 2:**
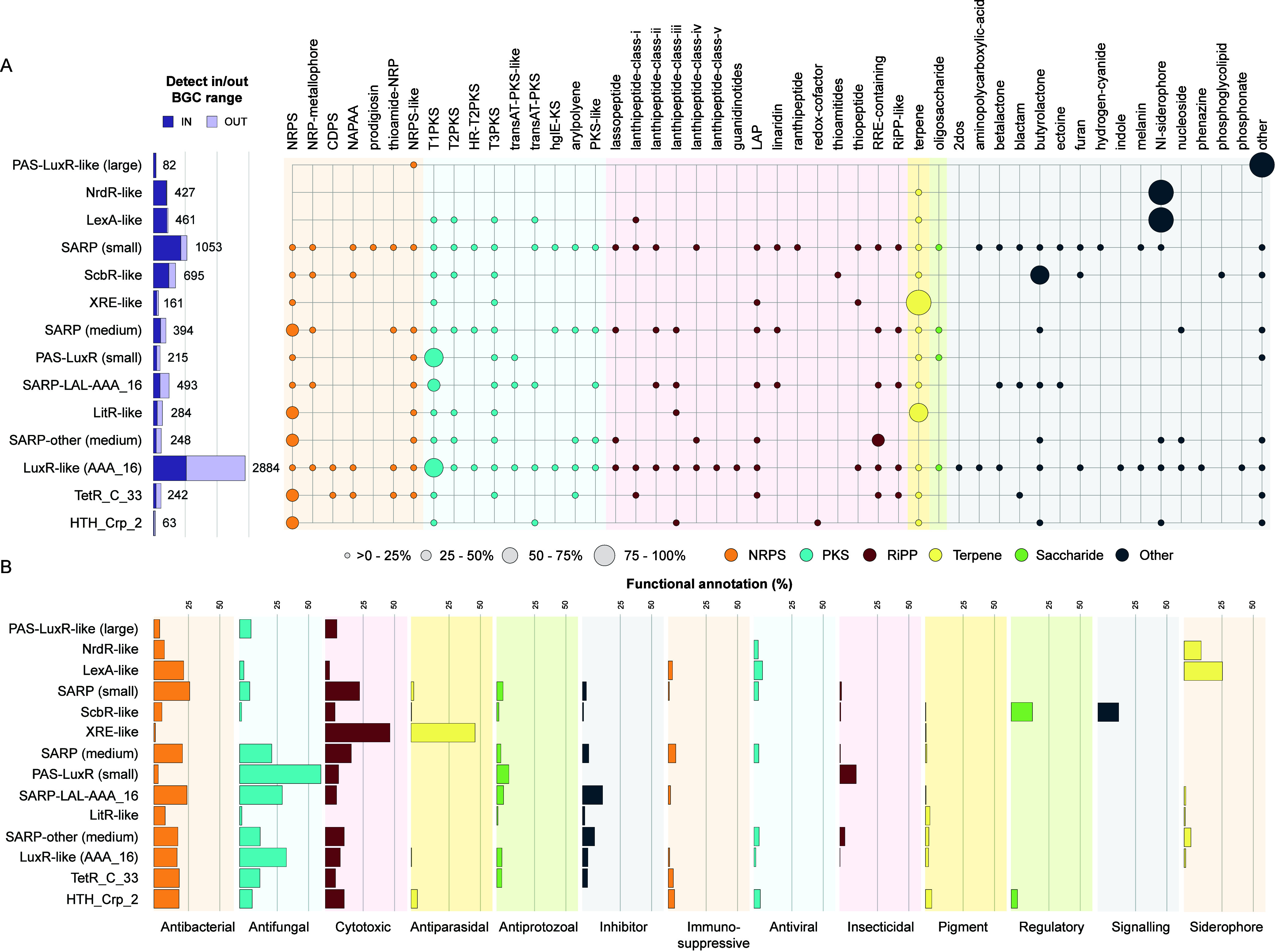
Functional assessment of regulatory subclasses most strongly associated with BGCs. (A) Analysis of the top 14 regulatory subclasses, displaying the proportion of hits within BGC regions and the classification of the nearest core gene. BGC classes are categorized into NRPSs, PKSs, RiPPs, terpenes, saccharides, and others, as defined by antiSMASH. Visualizations were generated using iTOL.^27^ (B) Functional assessment of regulators and associated BGCs, based on BGCs from the MIBiG v3.0 database.^28^ The detected BGCs must be at least 50% similar to a MIBiG BGC, and the regulator–BGC association must have been observed at least 10 times.

The resulting associations were further scrutinized to find matches that are associated with specific BGC classes, or even to specific gene cluster families (GCFs). Notably, this includes some of the regulatory genes with the highest in-BGC ratios, namely, large PAS-LuxR (97.6%), NrdR (95.6%), and LexA (91.8%). Genes encoding the large PAS-LuxR regulators are primarily found adjacent to genes recognized by antiSMASH’s bacilysin HMM profile (*n* = 79/82), which falls under the “other” category. This recognition indicates similarity to bacilysin-related ligases, which are associated with biosynthesis of the dipeptide antibiotic bacilysin. Genes encoding NrdR- and LexA-type regulators are commonly associated with nonribosomal peptide synthetase (NRPS)-independent (NI) siderophore BGCs. Further inspection revealed that genes encoding both NrdR and LexA regulators mainly co-occur within members of a GCF with unknown function (NrdR *n* = 352/427, LexA *n* = 360/461). Beyond these BGC-specific regulators, genes encoding XRE-like regulators (65.2% in-BGC ratio) were frequently observed within predicted terpene BGCs (*n* = 92/105 in-BGC), predominantly within the hopene cluster and its homologues (MIBiG BGC0000663, similarity >90%), although they are not strictly limited to this GCF. Similarly, genes encoding LitR-like regulators (42.3% in-BGC ratio) are also commonly associated with terpene BGCs (*n* = 66/120 in-BGC) but also frequently occur in NRPS-like clusters (*n* = 45/120 in-BGC).

To inform genome mining and prioritization of BGCs, a crucial question to answer is which regulatory genes may serve as beacons (or at least indicators) for BGCs with specific types of natural product chemistry or bioactive potential. Genes encoding SARP-family regulators are well represented with three of the four subgroups showing higher in-vs-out BGC ratios. The SARP subfamily that is most strongly associated with BGCs is that of the small SARPs (82.1%), followed by medium-sized SARPs (57.6%) and the SARP-LuxR hybrids (SARP-LALs) with the AAA ATPase domain AAA_16 (42.6%). Genes encoding small SARPs are found across various BGC classes, with the majority associated with genes for different types of polyketide synthases (PKSs, *n* = 440/864 in-BGC), while those encoding medium-sized SARPs are more commonly associated with NRPSs (*n* = 92/227 in-BGC) and ribosomally synthesized and post-translationally modified peptides (RiPPs, *n* = 74/227 in-BGC). In contrast, genes encoding SARP-LALs are more frequently linked to type I polyketide synthases (T1PKSs, *n* = 69/210 in-BGC). Additionally, genes encoding other LuxR family regulators, including the LuxR-AAA_16 (35.9% in-BGC ratio) and small PAS-LuxRs (48.8% in-BGC ratio), also tend to be more associated with BGCs for T1PKSs (*n* = 538/1034 and *n* = 76/105 in-BGC, respectively). To determine whether SARP regulator-BGC associations extend beyond *Streptomyces* species, we analyzed the presence of their genes across BGCs in the antiSMASH database, based on the occurrence of BTAD and trans_reg_c domains (Figure 1B). As expected, SARP genes are primarily found in *Streptomyces* sp. (68%), but also appear in other bacterial genera, such as *Paenibacillus* sp. (7%), *Lentzea* sp. (7%), and *Rhodococcus* sp. (6%). The distribution of regulator-BGC functions is similarly broad across classes, with T1PKS, terpenes, and NRPSs being the most common (Table S4). Genes encoding ScbR-like regulators of the TetR family (70.1% in-BGC ratio) are primarily connected to BGCs encoding butyrolactones (*n* = 347/487 in-BGC), small signaling molecules known to regulate morphological development and specialized metabolite production.^26^

Since some of these regulatory genes are associated with various BGC types, we investigated whether any are more specifically linked to certain BGC functions. To do this, we retrieved the known biological activity of the product of the most similar MIBiG BGC, if exceeding a similarity threshold of 50% (Figure 2B). For LitR, NrdR, ScbR, and TetR_C_33, the proportions of regulatory gene-containing BGCs without MIBiG-based associations with a known function were 84.3, 75.8, 49.8, and 47.8%, respectively. For those BGCs that could be connected to a known function, we observed that, although several LuxR family regulators were associated with T1PKS BGCs, the specific functions of these BGCs varied. Genes encoding small PAS-LuxRs were most frequently linked to BGCs specifying antifungals, while SARP-LALs and LuxR-AAA_16 were also associated with antibacterial activity.

### Regulators as Markers for Identifying Novel Biosynthetic Gene Clusters

Next, we investigated whether regulatory genes with a high in-BGC ratio could serve as beacons for potentially novel BGCs. We focused on small SARP genes, as they not only have the highest in-BGC ratio after the cluster-specific regulators (82.1%, *n* = 864/1053) but also are associated with a diverse range of BGC types (Figure 2A) known to specify compounds with antibacterial, antifungal, or cytotoxic activity (Figure 2B). We specifically examined the 17.9% (*n* = 189) of small SARPs encoded outside the boundaries of BGCs belonging to known classes detected by antiSMASH. Since predicted BGC regions may not accurately represent the true boundaries, regulatory genes situated just beyond the edge of a BGC could still be functionally linked to that BGC. To account for this, we identified genetic regions at least one approximate BGC length (20 kb) away from any other gene cluster, resulting in 82 regions with a substantial distance from predicted BGCs. To explore structural similarities within these regions, we applied the clustering algorithm BiG-SCAPE,^29^ which grouped them into 49 singletons and 11 clusters of similar gene regions (Figure 3). The functional annotation of each candidate BGC was manually assessed to evaluate its potential involvement in secondary metabolism. To streamline this process, we quantified the number of biosynthesis-related PFAMs within each gene region, allowing us to prioritize gene clusters more likely associated with secondary rather than primary metabolism. Indeed, gene clusters with a higher abundance of biosynthetic PFAMs often displayed annotations linked to known biosynthetic functions, such as aminotransferases, methyltransferases, condensation domains, and P450 oxidoreductases. For a comprehensive overview of all BGC regions and detected biosynthetic related domains, we refer to Table S5.

**Figure 3:**
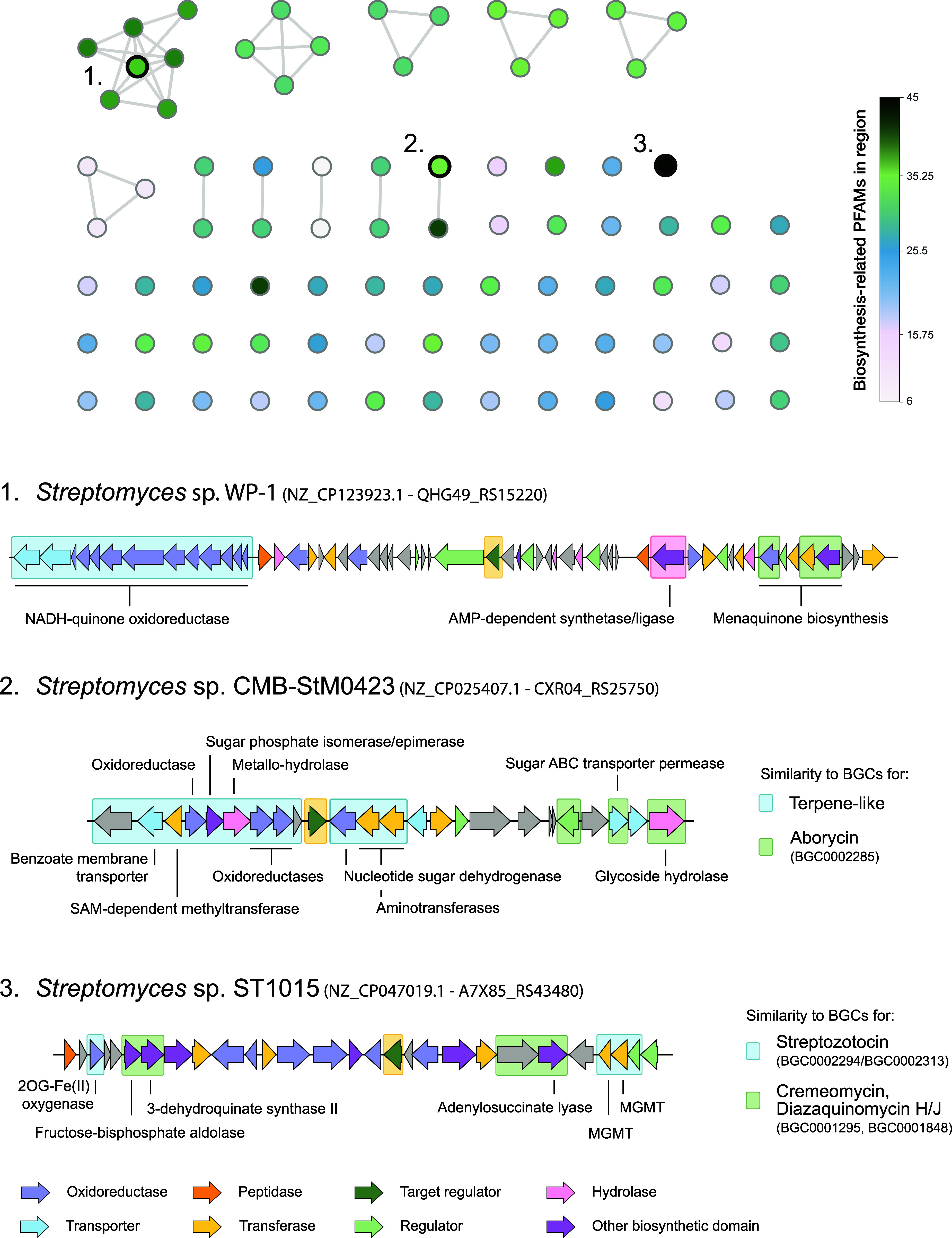
Cluster similarity network of candidate cluster regions containing a small SARP encoding gene. In the network on the top, each node represents a single cluster region, while edges are defined by the BiG-SCAPE^29^ similarity clustering algorithm, linking BGCs with high similarity. Node colors indicate the number of biosynthetic-associated Pfams detected within each candidate BGC. At the bottom, three selected BGCs are explored in detail, with colors representing the annotations of different enzyme classes within the genes. The target SARP is indicated within a yellow box.

We have highlighted three candidate BGCs to demonstrate the potential of using small SARPs as markers for discovering and prioritizing novel BGCs, including those not detectable by rule-based methods (Figure 3). The first region (NZ_CP123923.1) features genes encoding the subunits of NADH quinone oxidoreductase, an enzyme that catalyzes the reduction of quinones to hydroquinones, as well as various genes associated with menaquinone biosynthesis, a pathway known to encode potential antimicrobial drug targets. We also observed a gene encoding an AMP-dependent synthetase/ligase (QHG49_RS15290) that has hits to protein-coding genes from three BGCs in MIBiG (BGC0000595, BGC0000596, and BGC0000597) with similarity scores of 56, 56, and 51%, respectively. These hits correspond to BGCs for RiPPs, many of which encode antibiotics. In addition, we identified a double-repeat sequence (TTGCAGT-N10-TTGCAGT) matching the SARP binding pattern upstream of a putative menaquinone biosynthesis gene (QHG49_RS15195) using *de novo* motif discovery, suggesting likely control of this operon by a SARP.

The genes surrounding the small SARP regulatory gene in the second region (NZ_CP025407.1), spanning from CXR04_RS25705 to CXR04_RS25765, show over 50% ClusterBlast similarity in antiSMASH to several terpene clusters. These include genes encoding oxidoreductases, transferases, and a transporter, although the region lacks the core genes required for antiSMASH detection. Upstream of this operon, three genes, for a LacI regulator (CXR04_RS25800), an ABC transporter (CXR04_RS25810), and a glycoside hydrolase (CXR04_RS25820), respectively, revealed a combined similarity score of 52% to the aborycin BGC (BGC0002285), a type I lasso peptide RiPP.

The third region (NZ_CP047019.1) contains several enzyme-coding genes commonly associated with secondary metabolism and that show sequence similarity to genes from known BGCs, including those for cremeomycin (BGC0001295) and diazaquinomycin H/J (BGC0001848). Specifically, it includes a gene for a fructose-bisphosphate aldolase (A7X85_RS43425), which shows 63 and 49% BlastP identity to protein-coding genes from the cremeomycin and diazaquinomycin clusters, respectively, as well as to a gene for a 3-dehydroquinate synthase II (A7X85_RS43430), with 52 and 51% amino acid identity. Both of these genes also exhibit 51% identity to two of the three genes from a benzoate-forming subcluster of the platensimycin cluster (FJ655920). Additionally, there is similarity to genes for an FAD/NAD­(P)-binding protein (A7X85_RS43500) with 64 and 61% identity, and an adenylosuccinate lyase gene (A7X85_RS43505) showing 68 and 64% identity. Furthermore, genes within this region show similarity to genes from two streptozotocin BGCs (BGC0002294 and BGC0002313), including a 2-oxoglutarate (2OG) and Fe­(II)-dependent oxygenase-like gene (A7X85_RS43410, 58% identity), two O6-methylguanine-DNA methyltransferase (MGMT) genes (A7X85_RS43515 and A7X85_RS43520) with 55 and 65% identity, respectively, and a sigma factor gene (A7X85_RS43525, 69% identity), suggesting that the region is likely to encode a secondary metabolic pathway.

We repeated the process for the small PAS-LuxRs (Figure S2 and Table S6). The clustering resulted in 14 clusters and 23 singletons, of which all GCFs displayed ClusterBlast similarity with PKSs, particularly type I and type II PKSs. In contrast to SARP genes, which are associated with a broader range of BGC types, PAS-LuxR detection predominantly identifies PKS regions, which could be used to prioritize novel PKSs for experimental characterization.

## Discussion

In this work, we analyzed regulatory genes not only based on their general annotations but more extensively on their domain architecture using a protein domain co-occurrence (DCN) strategy.^30^ This approach allowed us to group regulatory genes into families and further refine them into subfamilies, which we then associated with their presence within predicted BGC ranges. This information can, in turn, be used to prioritize BGCs that contain a regulatory gene known to be more associated with a specific BGC class or functional classification. For instance, to identify terpene-associated clusters, we show that regions with XRE-type regulatory genes may be of interest. Similarly, when targeting antifungal compounds, regions containing small-sized PAS-LuxR genes could be explored, which agrees with previous work on antifungal compound discovery.^31,32^ However, it is important to note that many of the predicted BGCs lack functional annotations from the MIBiG database, which currently limits the use of functional annotations and bioactivities as a means to prioritize BGCs.

The refinement into subfamilies of regulatory genes demonstrates that, rather than studying regulatory families as a whole, studying these subfamilies results in better associations. This aligns with previous speculations about SARPs, where mainly representatives of the small SARP subfamily are considered promising targets for activation approaches.^22,25^ Indeed, we identified several subgroups of SARPs among those that are most frequently associated with BGCs, with small SARPs having the highest in-BGC ratio, reaffirming their importance for BGC regulation in *Streptomyces* species. In addition to small SARPs, we propose medium SARPs and SARP-LALs encoding genes as promising targets for further exploration, based on their high prevalence within predicted BGC regions. Aside from these SARPs, we also included an “other” category, which consists of regulators that may not follow the typical domain structure, such as those missing one of the common domains. This could be due to currently unknown and unidentified domains within these genes, suggesting that future improvements in domain identification could lead to better classifications, not only for this regulatory gene family but for all regulatory genes.

Current methods for BGC prioritization and discovery often focus on specific gene markers, such as the decRiPPter algorithm,^9^ which detects precursor peptides in RiPP BGCs, and the ARTS approach,^33^ which prioritizes BGCs based on self-resistance markers within the clusters. Here, we hypothesize that regions containing small SARPs, which remain undetected by current rule-based BGC prediction algorithms, may represent novel BGC regions and could serve as additional markers for their discovery. These rule-based methods are grounded in experimentally validated BGCs, which rules out noncanonical BGCs encoding yet-undiscovered biosynthetic pathway types. Accordingly, we demonstrate that targeting small SARP genes outside the predicted BGC boundaries can serve as an effective strategy for identifying novel noncanonical BGC candidates. We applied the same approach to regions containing PAS-LuxR genes and identified several novel, previously undetected PKS-like regions, further demonstrating that this method can be a valuable addition to BGC detection. The HMM profiles and scripts from this study can be used to identify regulator-BGC associations in *Streptomyces* and beyond, integrating this regulatory-focused approach into custom workflows. To ensure that these novel BGC candidates are not artifacts of genomic degradation, it is important to assess the conservation of the regions, particularly the operons within them, across multiple species. Conserved regions are far less likely to be undergoing degradation or loss, making them more likely candidates for novel BGCs. Given that the detection boundaries are arbitrarily set and the regulatory gene may not be part of the same operon, integrating additional data types could help pinpoint the exact BGC. For instance, transcription factor binding site (TFBS) predictions or de novo TFBS discovery,^34,35^ accounting for the autoregulation of target genes,^36^ could help identify the precise regulon of the regulatory gene. Supplementing this with coexpression data could further refine cluster boundaries and provide insights into expression conditions.^37,38^ Ultimately, this integrative strategy, leveraging multiple markers and data types, will provide a more comprehensive and sophisticated approach to BGC detection and prioritization.

Finally, artificial intelligence will likely lead to the identification of a large number of yet unknown gene cluster families (GCFs) for novel bioactive compounds. For large GCFs with many BGCs, an important strategy for rapid assessment of the biosynthetic potential will be to analyze at least a few BGCs, each associated with different regulatory gene families. This would make it likely that they are expressed under different conditions and thus increase the chances that the molecular product will be observed for at least one of them. As an example, recently a new GCF was identified for RiPPs (class V), which encodes novel modifying enzymes. Of the two BGCs analyzed within this GCF, the SARP-associated BGC for cacoidin was highly expressed,^39^ while that for pristinin that only contained a LuxR regulatory gene was near-silent.^9^

## Conclusions

Advances in experimental and computational methods have led to a substantial increase in the number of available bacterial genomes and predicted BGCs within them. With this abundance of clusters, there is great potential for the discovery of novel natural products, yet it also raises the challenge of how to prioritize BGCs for experimental validation and prevent the rediscovery of known compounds.^13^ Current genome mining tools typically rely on either rule-based approaches, which are grounded in experimentally characterized compounds and thus provide high reliability but are limited by the availability of such data, or machine learning-based methods, which can identify many potentially novel clusters but also generate a higher number of false positives.^40,41^ In this work, we investigated whether regulatory genes could aid in the prioritization of BGCs with bioactivities of interest, aiming to enhance BGC detection algorithms and provide researchers with a more effective way to identify clusters for experimental validation. Our findings show that the detection of specific regulatory genes can not only aid in the prioritization of BGCs with targeted functions but also reveal the potential for discovering many more previously undetected BGCs. Thus, we anticipate that incorporating regulatory predictions will become a crucial component in the effective detection, prioritization, and expression of BGCs, streamlining the discovery of novel natural products with bioactive potential and beyond.

## Methods

### Strain Collection

A total of 440 complete *Streptomyces* genomes were downloaded from the NCBI database in August 2023. Accession numbers and strain identifiers are listed in Table S1.

### Regulatory Protein Domain Detection

To identify regulatory-associated coding regions, we extracted 1375 profile hidden Markov models (pHMMs) from PFAM v36.0 ^42^ using 27 regulation-focused keywords. These keywords included structural identifiers such as “helix–turn–helix (HTH)” and “helix–loop–helix (HLH)”, as well as general identifiers like “repressor”, “activator”, and “DNA binding”. An overview of the keywords can be found in Table S2. A total of 128,993 regions matched the regulatory pHMM selection by using hmmsearch of HMMER v3.3.2 (https://hmmer.org/) with the gathering (GA) threshold. Each corresponding protein sequence was then searched against the entire PFAM library to generate a detailed overview of all present domains. Overlapping hits were filtered by selecting those with the highest normalized bitscore according to the PFAM profile cutoff.

### Domain Co-Occurrence Network Construction

For each protein, all coexisting domains were counted and used to construct a network using NetworkX v3.3,^43^ where nodes represent the domains and edges represent their co-occurrence within a single protein. Only co-occurrences detected at least a hundred times were retained to obtain the most common association patterns. The cluster algorithm MCL v14–137 ^44^ was applied with an inflation threshold score of 6, and the resulting network was visualized by Cytoscape v3.10.2.^45^

### Associating Protein Domains with Biosynthetic Gene Clusters

Gene clusters were detected in the *Streptomyces* collection using the prediction software antiSMASH v7.1.0 ^46^ with detection strictness “relaxed”. A custom version of the multiSMASH^47^ workflow was utilized to extract the BGC ranges and annotations for each BGC. Each protein domain was then compared to these BGC ranges to calculate the ratio of hits within or outside the range. For hits located within a BGC range, the corresponding BGC class and any available functional annotations were noted. This process was repeated for protein domain combinations, identified through co-occurrence network subclusters (Table S2). The functional assessment of regulators was based on annotations from the MIBiG v3.0 database.^28^ To minimize false positives, we filtered out BGCs with less than 50% similarity to a MIBiG cluster and removed associations observed fewer than 10 times. Conservation of SARP regulators was determined by searching the antiSMASH v4 ^48^ database for PFAM PF03704.21 and PF00486.32 using cblaster v1.3.18 ^49^ with the -u 2 -mh 1 -g 1 flags.

### Candidate BGC Detection and Clustering

The candidate BGCs were identified by selecting all small SARPs located outside the antiSMASH-predicted BGC boundaries, with a minimum distance of 20 kb from the predicted BGC borders. The target genes were then side-loaded into antiSMASH v7.1.0 ^46^ using the --sideload-by-cds flag, which generates subregions around specified locus tags with a default size of 20 kb. These sideloaded regions were used as input for the BiG-SCAPE v1.1.8 ^29^ clustering algorithm. The resulting networks were visualized using Cytoscape v3.10.2.^45^

## Supplementary Material





## Data Availability

All analysis scripts and data generated in this study are available at https://github.com/HAugustijn/regulator_profiling/

## Notes

Ratio of regulatory genes and CDSs within BGCs (Figure S1) and cluster similarity network of small PAS-LuxRs (Figure S2) (PDF)
Genomes (Table S1); domain detection (Table S2); regulatory families (Table S3); SARP family distribution (Table S4); SARP biosynthetic domains (Table S5); and LuxR biosynthetic domains (Table S6) (XLSX)

## Notes

Published as part of *Biochemistry* special issue “A Tribute to Christopher T. Walsh”.

## †

Z.L.R: Department of Ecology, Evolution and Marine Biology, University of California, Santa Barbara, California 93117, United States

## References

[1] Davies J., Davies D. (2010). Origins and Evolution of Antibiotic Resistance. Microbiol. Mol. Biol. Rev..

[2] Larsson D. G. J., Flach C.-F. (2022). Antibiotic Resistance
in the Environment. Nat. Rev. Microbiol..

[3] Walesch S., Birkelbach J., Jézéquel G., Haeckl F. P. J., Hegemann J. D., Hesterkamp T., Hirsch A. K. H., Hammann P., Müller R. (2023). Fighting Antibiotic Resistance-Strategies and (Pre)­Clinical
Developments to Find New Antibacterials. EMBO
Rep..

[4] Bernal F. A., Hammann P., Kloss F. (2022). Natural Products
in Antibiotic Development:
Is the Success Story Over?. Curr. Opin. Biotechnol..

[5] Hopwood, D. A. Streptomyces in Nature and Medicine. Oxford University PressNew: York, NY,February 3, 2007. 10.1093/oso/9780195150667.001.0001.

[6] Barka E. A., Vatsa P., Sanchez L., Gaveau-Vaillant N., Jacquard C., Klenk H. P., Clément C., Ouhdouch Y., van Wezel G. P. (2016). Taxonomy, Physiology, and Natural
Products of Actinobacteria. Microbiol. Mol.
Biol. Rev..

[7] Ziemert N., Alanjary M., Weber T. (2016). The Evolution
of Genome Mining in
Microbes - a Review. Nat. Prod. Rep..

[8] Cimermancic P., Medema M. H., Claesen J., Kurita K., Wieland
Brown L. C., Mavrommatis K., Pati A., Godfrey P. A., Koehrsen M., Clardy J., Birren B. W., Takano E., Sali A., Linington R. G., Fischbach M. A. (2014). Insights
into Secondary Metabolism from a Global Analysis of Prokaryotic Biosynthetic
Gene Clusters. Cell.

[9] Kloosterman A. M., Cimermancic P., Elsayed S. S., Du C., Hadjithomas M., Donia M. S., Fischbach M. A., van Wezel G. P., Medema M. H. (2020). Expansion of RiPP Biosynthetic Space through Integration
of Pan-Genomics and Machine Learning Uncovers a Novel Class of Lanthipeptides. PLoS Biol..

[10] Torres M. D. T., Brooks E. F., Cesaro A., Sberro H., Gill M. O., Nicolaou C., Bhatt A. S., de la Fuente-Nunez C. (2024). Mining Human
Microbiomes Reveals an Untapped Source of Peptide Antibiotics. Cell.

[11] Scherlach K., Hertweck C. (2021). Mining and Unearthing Hidden Biosynthetic Potential. Nat. Commun..

[12] Tran P. N., Yen M.-R., Chiang C.-Y., Lin H.-C., Chen P.-Y. (2019). Detecting
and Prioritizing Biosynthetic Gene Clusters for Bioactive Compounds
in Bacteria and Fungi. Appl. Microbiol. Biotechnol..

[13] van
Bergeijk D. A., Terlouw B. R., Medema M. H., van Wezel G. P. (2020). Ecology
and Genomics of Actinobacteria: New Concepts for Natural Product Discovery. Nat. Rev. Microbiol..

[14] Xu F., Wu Y., Zhang C., Davis K. M., Moon K., Bushin L. B., Seyedsayamdost M. R. (2019). A Genetics-Free
Method for High-Throughput Discovery
of Cryptic Microbial Metabolites. Nat. Chem.
Biol..

[15] Bode H. B., Bethe B., Höfs R., Zeeck A. (2002). Big Effects from Small
Changes: Possible Ways to Explore Nature’s Chemical Diversity. Chembiochem.

[16] Maglangit F., Fang Q., Kyeremeh K., Sternberg J. M., Ebel R., Deng H. (2020). A Co-Culturing Approach
Enables Discovery
and Biosynthesis of a Bioactive Indole Alkaloid Metabolite. Molecules.

[17] Meena S., Wajs-Bonikowska A., Girawale S., Imran M., Poduval P., Kodam K. (2024). High-Throughput Mining of Novel Compounds
from Known Microbes: A
Boost to Natural Product Screening. Molecules.

[18] Libis V., MacIntyre L. W., Mehmood R., Guerrero L., Ternei M. A., Antonovsky N., Burian J., Wang Z., Brady S. F. (2022). Multiplexed
Mobilization and Expression of Biosynthetic Gene Clusters. Nat. Commun..

[19] van
der Heul H. U., Bilyk B. L., McDowall K. J., Seipke R. F., van Wezel G. P. (2018). Regulation of Antibiotic Production in Actinobacteria:
New Perspectives from the Post-Genomic Era. Nat. Prod. Rep..

[20] McLean T. C., Wilkinson B., Hutchings M. I., Devine R. (2019). Dissolution of the
Disparate: Co-Ordinate Regulation in Antibiotic Biosynthesis. Antibiotics (Basel).

[21] Makitrynskyy R., Ostash B., Tsypik O., Rebets Y., Doud E., Meredith T., Luzhetskyy A., Bechthold A., Walker S., Fedorenko V. (2013). Pleiotropic
Regulatory Genes BldA,
AdpA and AbsB Are Implicated in Production of Phosphoglycolipid Antibiotic
Moenomycin. Open Biol..

[22] Yan Y., Xia H. (2024). The Roles of SARP Family
Regulators Involved in Secondary Metabolism
in Streptomyces. Front. Microbiol..

[23] Mingyar E., Mühling L., Kulik A., Winkler A., Wibberg D., Kalinowski J., Blin K., Weber T., Wohlleben W., Stegmann E. (2021). A Regulator Based “Semi-Targeted”
Approach
to Activate Silent Biosynthetic Gene Clusters. Int. J. Mol. Sci..

[24] Ye S., Molloy B., Pérez-Victoria I., Montero I., Braña A. F., Olano C., Arca S., Martín J., Reyes F., Salas J. A., Méndez C. (2023). Uncovering
the Cryptic Gene Cluster Ahb for 3-Amino-4-Hydroxybenzoate Derived
Ahbamycins, by Searching SARP Regulator Encoding Genes in the Streptomyces
Argillaceus Genome. Int. J. Mol. Sci..

[25] Krause J., Handayani I., Blin K., Kulik A., Mast Y. (2020). Disclosing
the Potential of the SARP-Type Regulator PapR2 for the Activation
of Antibiotic Gene Clusters in Streptomycetes. Front. Microbiol..

[26] Creamer K. E., Kudo Y., Moore B. S., Jensen P. R. (2021). Phylogenetic Analysis
of the Salinipostin γ-Butyrolactone Gene Cluster Uncovers New
Potential for Bacterial Signalling-Molecule Diversity. Microb. Genom..

[27] Letunic I., Bork P. (2024). Interactive Tree of
Life (ITOL) v6: Recent Updates to the Phylogenetic
Tree Display and Annotation Tool. Nucleic Acids
Res..

[28] Terlouw B. R., Blin K., Navarro-Mũoz J.
C., Avalon N. E., Chevrette M. G., Egbert S., Lee S., Meijer D., Recchia M. J. J., Reitz Z. L., van Santen J. A., Selem-Mojica N., Tørring T., Zaroubi L., Alanjary M., Aleti G., Aguilar C., Al-Salihi S. A. A., Augustijn H. E., Avelar-Rivas J. A., Avitia-Domínguez L. A., Barona-Gómez F., Bernaldo-Agüero J., Bielinski V. A., Biermann F., Booth T. J., Carrion Bravo V. J., Castelo-Branco R., Chagas F. O., Cruz-Morales P., Du C., Duncan K. R., Gavriilidou A., Gayrard D., Gutiérrez-García K., Haslinger K., Helfrich E. J. N., van der Hooft J. J. J., Jati A. P., Kalkreuter E., Kalyvas N., Kang K. B., Kautsar S., Kim W., Kunjapur A. M., Li Y. X., Lin G. M., Loureiro C., Louwen J. J. R., Louwen N. L. L., Lund G., Parra J., Philmus B., Pourmohsenin B., Pronk L. J. U., Rego A., Rex D. A. B., Robinson S., Rosas-Becerra L. R., Roxborough E. T., Schorn M. A., Scobie D. J., Singh K. S., Sokolova N., Tang X., Udwary D., Vigneshwari A., Vind K., Vromans S. P. J. M., Waschulin V., Williams S. E., Winter J. M., Witte T. E., Xie H., Yang D., Yu J., Zdouc M., Zhong Z., Collemare J., Linington R. G., Weber T., Medema M. H. (2023). MIBiG 3.0:
A Community-Driven Effort to Annotate Experimentally Validated Biosynthetic
Gene Clusters. Nucleic Acids Res..

[29] Navarro-Muñoz J. C., Selem-Mojica N., Mullowney M. W., Kautsar S. A., Tryon J. H., Parkinson E. I., De Los Santos E. L. C., Yeong M., Cruz-Morales P., Abubucker S., Roeters A., Lokhorst W., Fernandez-Guerra A., Cappelini L. T. D., Goering A. W., Thomson R. J., Metcalf W. W., Kelleher N. L., Barona-Gomez F., Medema M. H. (2020). A Computational
Framework to Explore Large-Scale Biosynthetic Diversity. Nat. Chem. Biol..

[30] Wang Z., Zhang X.-C., Le M. H., Xu D., Stacey G., Cheng J. (2011). A Protein Domain Co-Occurrence Network
Approach for Predicting Protein
Function and Inferring Species Phylogeny. PLoS
One.

[31] Santos-Aberturas J., Payero T. D., Vicente C. M., Guerra S. M., Cañibano C., Martín J. F., Aparicio J. F. (2011). Functional Conservation
of PAS–LuxR
Transcriptional Regulators in Polyene Macrolide Biosynthesis. Metab. Eng..

[32] Han X., Liu Z., Zhang Z., Zhang X., Zhu T., Gu Q., Li W., Che Q., Li D. (2017). Geranylpyrrol A and
Piericidin F
from Streptomyces Sp. CHQ-64 ΔrdmF. J.
Nat. Prod..

[33] Mungan M. D., Alanjary M., Blin K., Weber T., Medema M. H., Ziemert N. (2020). ARTS 2.0: Feature Updates and Expansion
of the Antibiotic
Resistant Target Seeker for Comparative Genome Mining. Nucleic Acids Res..

[34] Augustijn H. E., Roseboom A. M., Medema M. H., van Wezel G. P. (2024). Harnessing
Regulatory Networks in Actinobacteria for Natural Product Discovery. J. Ind. Microbiol. Biotechnol..

[35] Rigali S., Anderssen S., Naômé A., van Wezel G. P. (2018). Cracking
the Regulatory Code of Biosynthetic Gene Clusters as a Strategy for
Natural Product Discovery. Biochem. Pharmacol..

[36] Anderssen S., Naômé A., Jadot C., Brans A., Tocquin P., Rigali S. (2022). AURTHO: Autoregulation
of Transcription Factors as
Facilitator of Cis-Acting Element Discovery. Biochim. Biophys. Acta Gene Regul. Mech..

[37] Augustijn H. E., Reitz Z. L., Zhang L., Boot J. A., Elsayed S. S., Challis G. L., Medema M. H., van Wezel G. P. (2024). Prediction
of Gene Cluster Function Based on Transcriptional Regulatory Networks
Uncovers a Novel Locus Required for Desferrioxamine B Biosynthesis. bioRxiv.

[38] Lee Y., Choe D., Palsson B. O., Cho B. (2024). Machine-Learning Analysis
of Streptomyces Coelicolor Transcriptomes Reveals a Transcription
Regulatory Network Encompassing Biosynthetic Gene Clusters. Adv. Sci. (Weinh.).

[39] Ortiz-López F. J., Carretero-Molina D., Sánchez-Hidalgo M., Martín J., González I., Román-Hurtado F., de la Cruz M., García-Fernández S., Reyes F., Deisinger J. P., Müller A., Schneider T., Genilloud O. (2020). Cacaoidin,
First Member of the New Lanthidin RiPP Family. Angew. Chem., Int. Ed. Engl..

[40] Arnold A., Alexander J., Liu G., Stokes J. M. (2023). Applications of
Machine Learning in Microbial Natural Product Drug Discovery. Expert Opin. Drug Discovery.

[41] Medema M. H., Fischbach M. A. (2015). Computational
Approaches to Natural Product Discovery. Nat.
Chem. Biol..

[42] Mistry J., Chuguransky S., Williams L., Qureshi M., Salazar G. A., Sonnhammer E. L. L., Tosatto S. C. E., Paladin L., Raj S., Richardson L. J., Finn R. D., Bateman A. (2021). Pfam: The Protein Families
Database in 2021. Nucleic Acids Res..

[43] Hagberg, A. A. ; Schult, D. A. ; Swart, P. J. Exploring Network Structure, Dynamics, and Function Using NetworkX. In Proceedings of the Python in Science Conference; SciPy, 2008; pp 11–15.

[44] Van
Dongen S. (2008). Graph Clustering via a Discrete Uncoupling Process. SIAM J. Matrix Anal. Appl..

[45] Shannon P., Markiel A., Ozier O., Baliga N. S., Wang J. T., Ramage D., Amin N., Schwikowski B., Ideker T. (2003). Cytoscape: A Software Environment
for Integrated Models
of Biomolecular Interaction Networks. Genome
Res..

[46] Blin K., Shaw S., Augustijn H. E., Reitz Z. L., Biermann F., Alanjary M., Fetter A., Terlouw B. R., Metcalf W. W., Helfrich E. J. N., van Wezel G. P., Medema M. H., Weber T. (2023). AntiSMASH
7.0: New and Improved Predictions for Detection, Regulation, Chemical
Structures and Visualisation. Nucleic Acids
Res..

[47] Reitz, Z. MultiSMASH v0.4.0; Zenodo, 2024. 10.5281/ZENODO.8276143.

[48] Blin K., Shaw S., Medema M. H., Weber T. (2024). The AntiSMASH Database
Version 4: Additional Genomes and BGCs, New Sequence-Based Searches
and More. Nucleic Acids Res..

[49] Gilchrist C. L. M., Booth T. J., van Wersch B., van Grieken L., Medema M. H., Chooi Y. H., Ouangraoua A. (2021). Cblaster:
A Remote Search Tool for Rapid Identification and Visualization of
Homologous Gene Clusters. Bioinform. Adv..

