# Supplementary material for: Regulatory Genes as Beacons for Discovery and Prioritization of Biosynthetic Gene Clusters in Streptomyces
Source: Biochemistry. 2025 Mar 26;64(13):2877–85. doi: 10.1021/acs.biochem.4c00711 (PMC12224297; doi:10.1021/acs.biochem.4c00711)
Supplement: Supplementary file 1 [file bi4c00711_si_001.pdf]

Supporting Information for Publication (SI)

Regulatory genes as beacons for discovery and prioritization of biosynthetic gene clusters in *Streptomyces*

Hannah E. Augustijn[1,2], Daan van Nassauw[1, #], Simona Cernat[2, #], Zachary L. Reitz[1,†], Gilles P. van Wezel[2,*], Marnix H. Medema[1,2,*]

[1] Bioinformatics Group, Wageningen University, 6708 PB, Wageningen, The Netherlands;

[2] Molecular Biotechnology, Institute of Biology, 2333 BE, Leiden University, Leiden, The Netherlands;

[#] Contributed equally

* Co-corresponding authors: g.wezel@biology.leidenuniv.nl, marnix.medema@wur.nl

[†] Z.L.R: Department of Ecology, Evolution and Marine Biology, University of California, Santa Barbara, CA 93117, USA

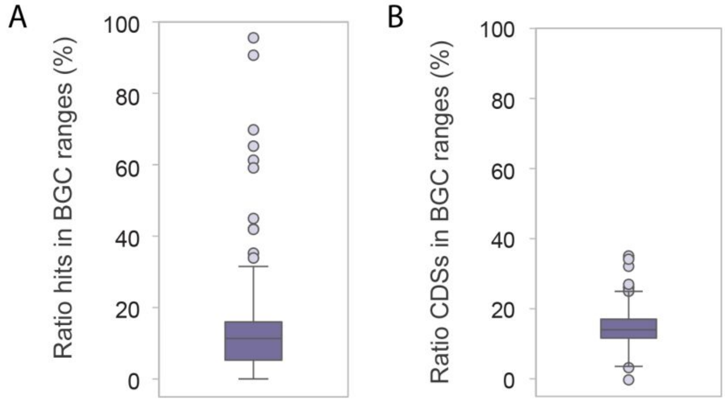

Figure S1. Ratio of regulatory genes and coding sequences (CDSs) within biosynthetic gene clusters (BGCs). A, For each regulatory gene family (n=279), the ratio of genes located within antiSMASH-predicted BGC regions versus those outside is visualized. Each data point represents the ratio for a specific regulatory gene family (e.g., small SARPs) within antiSMASH-predicted BGCs (n=14081) across the 440 tested *Streptomyces* species. **B,** Each dot represents the ratio of all CDSs of the 440 *Streptomyces* species located within antiSMASH-predicted BGCs (n=14081) versus outside.

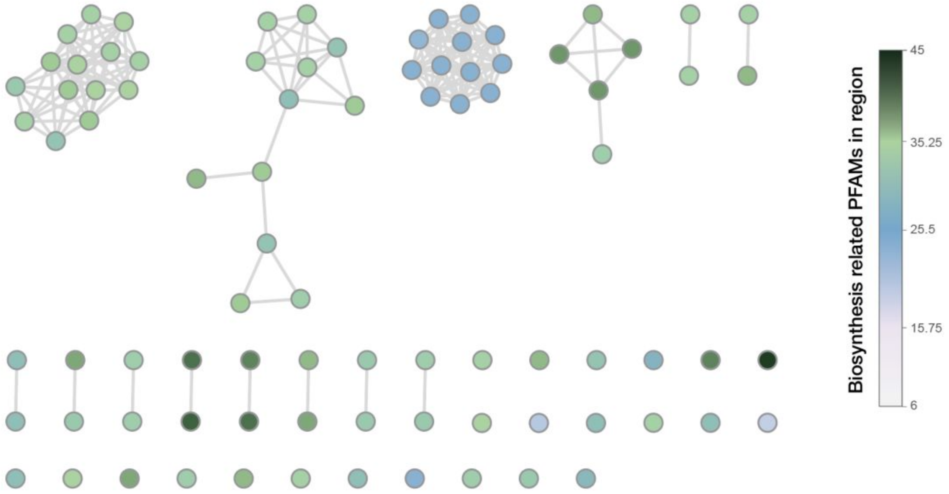

Figure S2. Cluster similarity network of candidate cluster regions containing a small PAS-LuxR encoding gene. Each node represents a single cluster region, while edges are defined by the BiG-SCAPE similarity clustering algorithm. Node colors indicate the number of biosynthetic-associated Pfams detected within each gene cluster.
